# Mr. D'Arcy Power's Evokement of "Bart.'s."

**Published:** 1918-03-23

**Authors:** 


### THE MUSE OF HOSPITAL HISTORY.

### Mr. D'Arcy Power's Evokement of "Bart.'s."

One of the most original ideas at the root of any modern book was conceived by Mr. Hilaire Belloc when he planned and wrote those sketches of historical events, presented from the point of view of some real or imagined actor in them, which he grouped together and called "The Eye-Witness." The same idea has inspired Mr. D'Arcy Power in penning the "Episodes in the History of the Hospital," St. Bartholomew's, of which a reprint lies before us from the hospital's *Journal*. It is also noteworthy that the source of inspiration in either case is seen to be a personal power of evoking landscape, an evokement indeed, since the dust of ages and the ruin of centuries obscure most of the landmarks of the past.

The source of delight in this form of writing arises from the vividness which makes each event seem to have been a real happening to the narrator, as if the reader were listening to one who has returned from a long journey or met with some experience which has left a lasting impression on his life. The dramatic form, especially the monologue, is therefore proper to such works, and the only flaw which we find in Mr. D'Arcy Power's papers is such an occasional intrusive phrase as "the vision changes," which breaks a spell that broods happily content when the transition from one incident to another is made more simply; for instance, by the mere phrase "the recollection which comes clearest to my mind." The imagination is not troubled by the fact that the teller of events at different epochs cannot be the same person, in time, at each. It cares only for the continuity of a form which suggests the eye-witness most naturally. A master of this form of narrative was William Morris, whose sense of the past was a "recollection" more than a re-creation, a gathering up of threads which other men had dropped, as, indeed, he did in reviving, at the high point from which they afterwards declined, the lost arts of printing, staining glass, weaving tapestry, and writing romances.

We have thought it well to linger on the style of these historical stories, because while the subject of them is familiar, the secret of their proper telling has been forgotten. Mr. D'Arcy Power has rendered, therefore, a treble service in these sketches—a service to his hospital by vivifying the bones in the valley of its past, to hospital literature by reminding men of a fine way in which to write it, and to general literature by the cultivation of a small plot of ground to much advantage. To have achieved so much in a pamphlet of twenty pages is a real success. The principle on which the success is based is as important as it has been neglected; but to recognise and welcome the principle is one thing; to be able to apply it is the gift of the historical muse.

